# Apalutamide and autophagy inhibition in a xenograft mouse model of human prostate cancer

**DOI:** 10.1007/s00432-022-04059-1

**Published:** 2022-06-25

**Authors:** Daniel Eberli, Benedikt Kranzbühler, Lukas Prause, Valentin Baumgartner, Sheryl Preda, Rosa Sousa, Fabienne Lehner, Souzan Salemi

**Affiliations:** Department of Urology, Laboratory for Urologic Oncology and Stem Cell Therapy, University Hospital Zürich, Wagistrasse 21, 8952 Schlieren, Switzerland

**Keywords:** Autophagy, Prostate cancer, Apalutamide, In vivo

## Abstract

**Background:**

Apalutamide (APA) is a next-generation androgen receptor antagonist for the treatment of advanced prostate cancer. We have previously shown that upregulation of autophagy is one of the mechanisms by which prostate cancer (PC) cells survive APA anti-tumor treatment in vitro. Therefore, we investigated the characteristics of the autophagic response to APA treatment, alone and in combination with autophagy inhibition, in an in vivo model.

**Methods:**

Tumor cells were injected into previously castrated nude mice. Four groups of mice bearing LNCaP xenografts were treated with daily intraperitoneal (i.p.) injections of vehicle (control), APA (10 mg/kg), APA (10 mg/kg) + Chl (Chloroquine, 10 mg/kg) or Chl (10 mg/kg). The animals of each treatment group (3/treatment) were kept for the duration of 2 and 3 weeks. At the end of the experiments, the animals were sacrificed and all samples assessed for tumor weight and size, histological analysis, immunoblotting (WES) and immunofluorescence.

**Results:**

The tumor weight was significantly reduced in mice treated with APA + Chl (203.2 ± 5.0, SEM, *P* = 0.0066) compared to vehicle control (380.4 ± 37.0). Importantly, the combined treatment showed a higher impact on tumor weight than APA (320.4 ± 45.5) or Chl (337.9 ± 35) alone. The mice treated with the combination of APA + Chl exhibited a reduced expression of ATG5 (autophagy-related five protein), Beclin 1 and LC3 punctuations and an increase in P62 as visualized by immunofluorescence and WES. In addition, Ki-67 nuclear staining was detected in all samples however reduced in APA + Chl (58%) compared to vehicle control (100%). The reduction in Ki-67 protein was associated with an increase in caspase 3 and endothelial CD31 protein expression.

**Conclusion:**

These data demonstrate that a treatment with APA + Chl leads to reduced autophagy levels and to tumor suppression compared to the APA monotherapy. Hence, the increased antitumor effect of APA in combination with autophagy inhibitors might provide a new therapeutic approach potentially translatable to patients.

## Introduction

Prostate cancer (PC) is the most common cancer among men worldwide. High-risk PC tends to recur in up to 40% of all patients following initial treatment (Lam et al. 2006). Despite second-line therapy, the majority of these patients develop castration-resistant prostate cancer (CRPC) with metastases, mainly in bone (Lam et al. 2006). Current guidelines recommend combined treatment with second generation of antiandrogens like apalutamide, enzalutamide, abiraterone or darolutamide which block the AR (androgen receptor) in patients with advanced PC (Beer et al. 2014; Ryan et al. 2013). However, some patients stop responding to these therapies due to drug resistance and castration resistance thus, the search for new AR-targeting compounds continues. Apalutamide (APA) is a potent AR inhibitor established for the treatment of advanced PC. It inhibits AR nuclear translocation, DNA binding and transcription of AR gene targets. Although APA and bicalutamide bind to the same AR–ligand binding domain, APA has a seven–tenfold greater affinity (Clegg et al. 2012). Phase I and II studies of APA reported a significant antitumor activity in patients with non-metastatic CRPC (Smith et al. 2016; Rathkopf et al. 2013). Together with the results of the SPARTAN trial, these findings led to the approval of apalutamide (Erleada™) for the treatment of non-metastatic CRPC (Smith et al. 2018).

Autophagy is a cellular self-digestive process controlling degradation of cellular contents and thereby essentially contributing to homeostasis (Mizushima and Komatsu 2011). Induction of autophagy is often detected in cancer cells during anti-cancer therapy, which involves severe metabolic changes and/or DNA damage (Fulda 2018). Dysregulation of autophagy in PC has been demonstrated by several research groups including ourselves (Kranzbuhler et al. 2019; Mortezavi et al. 2018; Nguyen et al. 2014). In our previous in vitro studies, we showed upregulation of ATG5 and autophagy in PC cell line. Moreover, we provided evidence showing that downregulation of ATG5 accelerates cell death and increases the efficacy of the anti-cancer drugs EPI-001, abiraterone acetate and APA (Kranzbuhler et al. 2019; Mortezavi et al. 2018; Eberli et al. 2020). In addition, we have shown that proteins related to autophagy are significantly upregulated in patients with advanced PC (Mortezavi et al. 2017). Given the promising antitumor effect of APA for the treatment of advanced PC and the role of autophagy as resistance mechanism against therapy (Nguyen et al. 2014), we aimed to investigate the level of autophagy in response to treatment with APA. Furthermore, we have previously shown that targeting autophagy—alone or in combination with its inhibitors—is effective at enhancing cell death (Eberli et al. 2020). Therefore, we believe that PC cells use autophagy to escape the insult of androgen deprivation or anti-androgen therapies as a survival mechanism. Hindering autophagy might be a way to overcome resistance mechanism toward therapy in CRPC. To confirm our in vitro results, we investigated the enhanced therapeutic effects of a combination treatment of APA with an autophagy inhibitor such as chloroquine (Chl) in a mouse xenograft model.

## Materials and methods

### Cell culture

PC cell line LNCaP (ATCC, CRL-1740) was purchased from American type culture collection (ATCC, Manassas, USA). Cells were cultivated in RPMI (Life Technologies, ThermoFisher Scientific, Waltham, MA, USA) supplemented with 10% FBS and 1% penicillin/streptomycin and incubated at 37 °C with 5% CO_2_. Medium was changed twice a week.

### Animal experimentation

All animal experiments were approved by the cantonal veterinary office (Veterinäramt Zürich, license No.244/2016) and performed according to the Swiss animal welfare act. A total of 28 male nude mice (8 weeks old; Charles River Laboratories, Sulzfeld, Germany) were analyzed. Mice underwent castration before tumor formation. After 2 weeks, nude mice were subcutaneously injected with 5.0 × 10^6^ LNCaP cells with a high concentration Matrigel carrier (500 µl, Corning Life Sciences, NY, USA) on both left and right backsides. Drug injection was started once the tumors were formed, 2–3 weeks after tumor cell injections. The mice were divided into four groups (three animals/group/ time point). The treatment groups Vehicle control (For APA; 18% PEG 400, 1% Tween 80, 1% polyvinyl pyrolidone, 65% 20 mM citrate buffer pH:4.0 in 0.5% carboxymethylcellulose sodium salt, all purchased from Sigma Aldrich), APA (10 mg/kg, Janssen Pharmaceutica NV, Belgium), APA (10 mg/kg) + Chl (10 mg/kg, Sigma-Aldrich, Buchs, Switzerland) and Chl (10 mg/kg) were subjected to intraperitoneal (i.p.) injections. Half of the animals of each treatment group (three/treatment) were kept for the duration of 2 and other half for 3 weeks. At the end of the experiments, animals were sacrificed and all samples were assessed for tumor weight and size.

### Tumor sample preparation and histological analysis

The tumor samples obtained from each mouse were divided into two pieces. One part was snap-frozen for gene and protein analysis. The second part of the tissue was fixed in 10% buffered formalin (Fisher Scientific, Norcross, GA), then processed and finally embedded in soft paraffin (Sargent-Welch Scientific, Skokie, IL). Paraffin sections were prepared (5 μm) and further processed. Haematoxylin and eosin (H&E, Sigma-Aldrich, Buchs, Switzerland) and Masson’s Trichrome (Sigma Aldrich, Buchs, Switzerland) staining were performed according to the manufacturer’s protocol.

### Immunofluorescent staining

Paraffin-embedded tumor samples were first de-paraffinized by treatment with xylene and then rehydrated by passage through a graded series of ethanol. The indirect immunostainings of tissue sections were performed at 4 °C overnight using the following primary antibodies for the autophagy-related proteins: anti-ATG5 (1:100, 0262-100, 7C6, nanoTools, Taningen, Germany), anti-Beclin 1 (1:200, NB110-87318, NanoTools, Taningen, Germany), LC3 (1:100, 0231–100, 5F10, nanoTools, Taningen, Germany), anti-Caspase 3, active (1:100, cleaved, AB3623, Merck, Switzerland), and anti-Ki-67 (1:100, AB9260, Merck, Switzerland). The slides were incubated with the secondary antibodies goat anti-mouse FITC (1:500, BD Biosciences Allschwil, Switzerland), goat anti-rabbit FITC (1:500, Vector Laboratories, Liestal, Switzerland) or Cy3-conjugated goat anti-mouse antibody (1:1000, Sigma Aldrich, Sigma Aldrich, Buchs, Switzerland) at room temperature for 1 h. Subsequently, they were counter-stained with DAPI (4′,6-diamidino-2-phenylindole, 1:200, Sigma Aldrich, Buchs, Switzerland). For negative controls, the primary antibody was omitted. Images were acquired with a Leica fluorescence microscope (CTR 6000).

### Immunoblotting (automated western blotting—WES)

The harvested tumor samples were pulverized in liquid nitrogen with a mortar/pestle and re-suspended in modified lysis buffer supplemented with a protease inhibitor cocktail (Sigma-Aldrich, Buchs, Switzerland). Samples were centrifuged for 20 min at 13,000 rpm and the supernatant was collected for protein determination. Total protein was measured using a BCA protein assay kit (Thermo scientific, Lausanne, Switzerland). Protein at 1 mg/mlL concentration was used for the WES using a 12–230 kDa cartridge kit (Protein Simple WES, Germany). Primary antibodies for autophagy-related proteins were mouse anti-ATG5 (1:100, NanoTools, Taningen, Germany), rabbit anti-Beclin1, rabbit anti-P62, and mouse anti-LC3B (all 1:50, Novus Biologicals Europe, Abingdon, United Kingdom). Mouse anti-GAPDH (1:100, Novus Biologicals Europe, Abingdon, United Kingdom) served as internal control. Samples were analyzed using the Compass software (ProteinSimple). Virtual blot and electropherogram of each sample was checked and evaluated. A sharply defined chemiluminescent signal was quantified by the software and the area of each sample was normalized to GAPDH.

### Statistical analysis

Results were analyzed by one-way ANOVA with Bonferroni’s post correction using GraphPad Prism (GraphPad Software, Inc., La Jolla, CA, version 7). *P *values < 0.05 were considered statistically significant. All data presented are expressed as means with corresponding standard error of the mean (± SEM).

## Results

### APA and autophagy inhibitor as potent tumor growth inhibitors in a humanized mouse xenograft model of CRPC

**Fig. 1:**
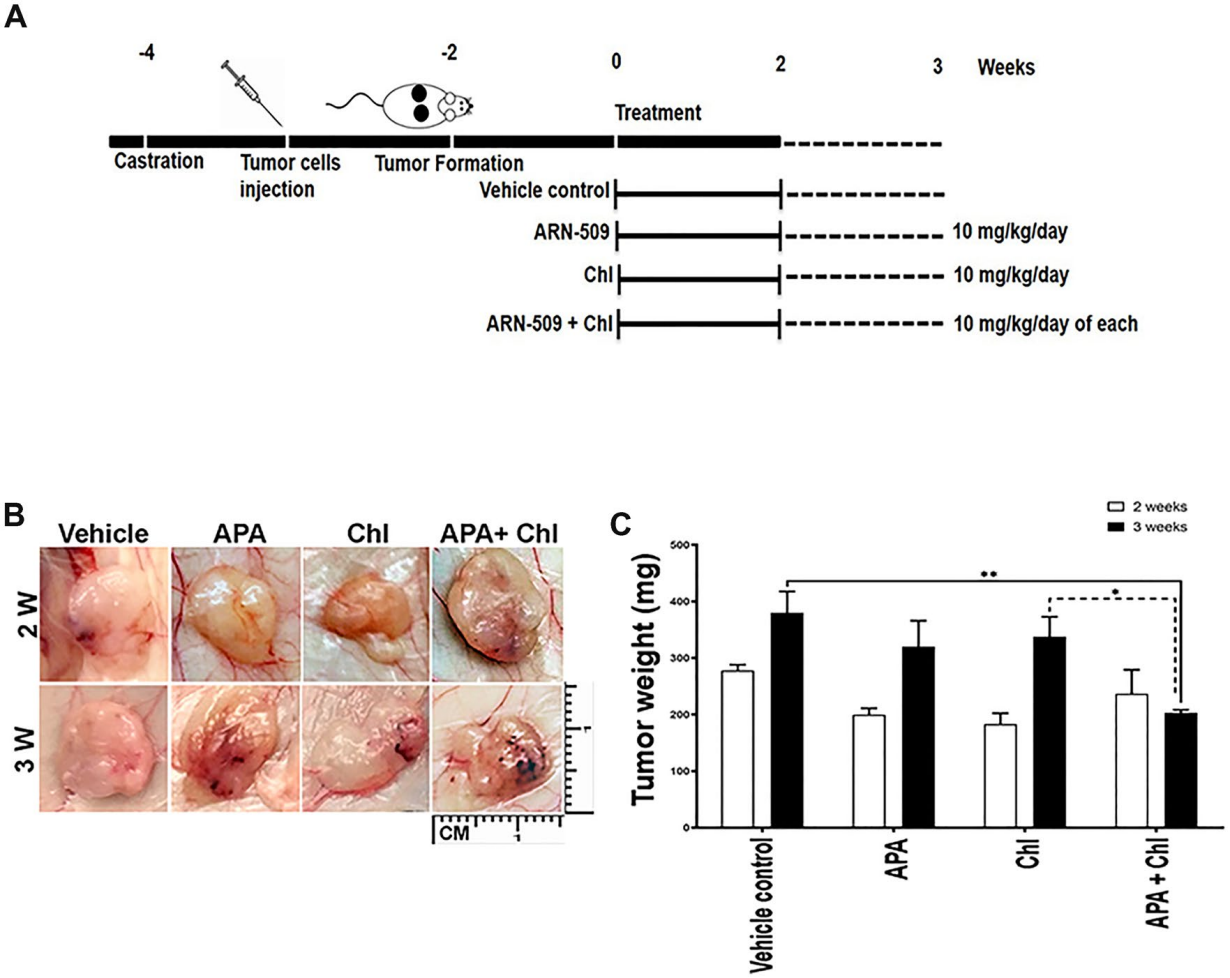
Effect of apalutamide (APA), hydroxyl chloroquine (Chl), and the combination of both in xenograft mouse model. **A** Schematic presentation of the animal study and injections time table. **B** In vivo tumor growth is shown with APA only, Chl only and combined treatment in a mouse xenograft model. LNCaP prostate cancer cells were injected subcutaneously to left and right backsides of castrated nude mice and grown for 2 weeks. Mice were treated 5 days per week for 2 and 3 weeks with vehicle control, APA (10 mg/kg), Chl (10 mg/kg), or both in combination (APA 10 mg/kg + Chl 10 mg/kg). **C** Total tumor weight following tissue harvesting after 2 and 3 weeks upon treatments. *N* = 6 per experimental condition

To confirm our previous in vitro findings (Eberli et al. 2020), to provide pre-clinical support and finally to test the impact of a combined treatment of APA with an autophagy inhibitor, we generated a PC xenograft model and analyzed the growth of LNCaP cells in vivo after inoculation of castrated male athymic nude mice (Fig. 1A). Primary tumor growth was observed in all injected mice (100%) (Fig. 1B). Drug injections (i.p.) were started once the tumors were formed, 3 weeks after tumor cell injection. The intraperitoneal treatment was applied for 5 days a week for up to 2 and 3 weeks with either vehicle control, APA (10 mg/kg), Chl (10 mg/kg) or Chl + APA (10 mg/kg). The animals did not reach the termination criteria after a mean growth period of 4 and 5 weeks (i.e., 2 and 3 weeks upon injections). The growth of the LNCaP xenograft was rapid in the control group (Fig. 1C) after 2 and 3 weeks. A decrease in tumor weight was detected under all experimental conditions compared to vehicle control after week 2 and 3. However, the most significant weight reduction was observed after 3 weeks with a significantly reduced tumor weight in mice treated with APA + Chl (203.2 ± 5.0, SEM, *P* = 0.0066) compared to vehicle control (380.4 ± 37.0). Importantly, the combined treatment had a higher impact on tumor weight than APA (320.4 ± 45.5) or Chl (337.9 ± 35) alone.

**Fig. 2:**
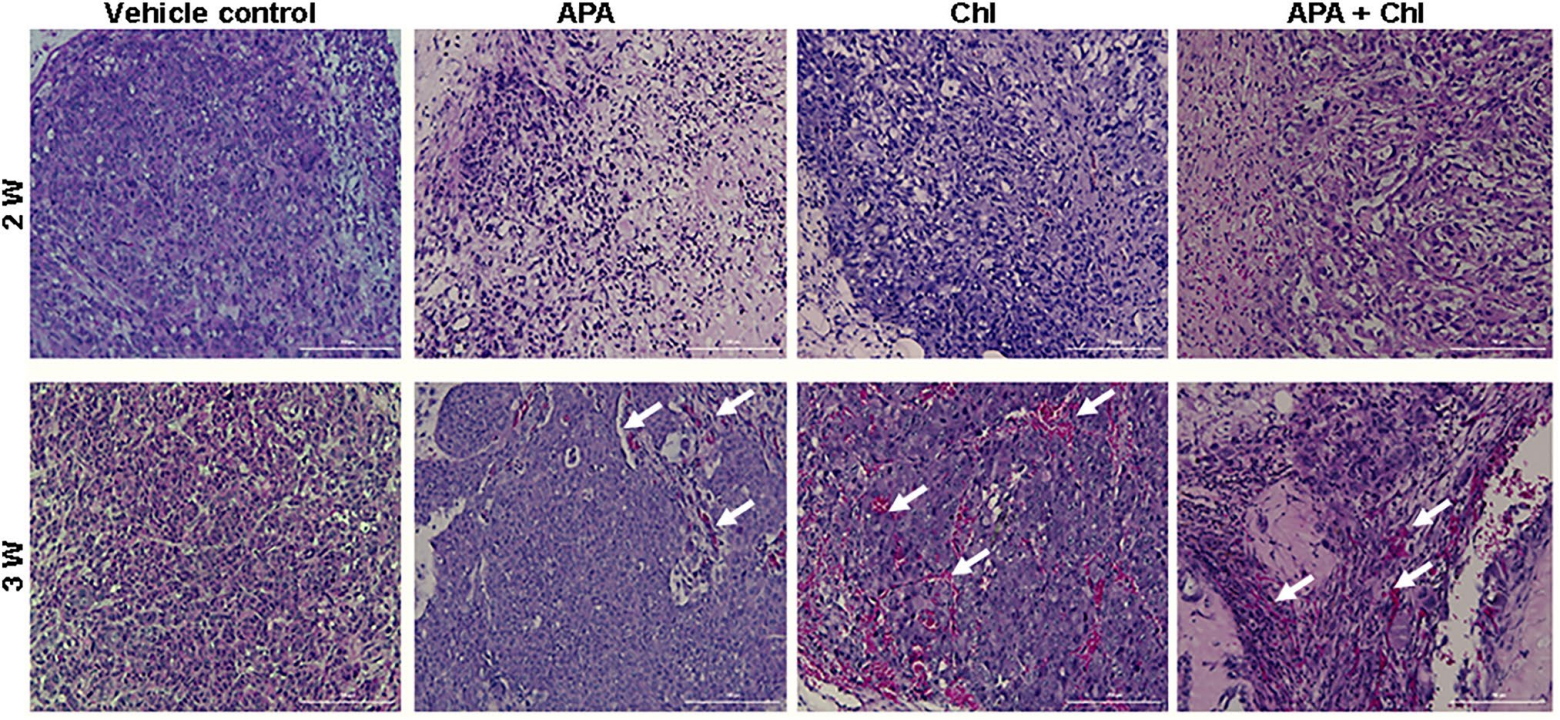
Representative images of paraffin-fixed hematoxylin and eosin-stained sections of formed tumor from all experimental conditions after 2 and 3 weeks of treatments with APA, Chl, and the combination of both. Hematoxylin stained the nuclei (blue–purple), eosin stained the cytoplasm and red blood cells in pink. Tumor tissue sections from combination treated animals with APA + Chl showed increased infiltration of inflammatory cells and reduced cell population density. Arrows (white) indicate increase in inflammatory cells

The average body weight of the animals did not vary significantly between treatment groups throughout the study. Importantly, there were no observable signs of stress or infection, such as redness or swelling at the injection site, change in behavior or activity in the three animal groups receiving the APA, Chl or the APA + Chl treatment compared to vehicle control. This finding suggests that APA + Chl has an anti-tumor activity against human prostate cancer in vivo, and is well-tolerated by mice at the dosage tested. Histological examination of the tumors showed increased infiltration of inflammatory cells in the combination treatment of APA + Chl compared with vehicle control, APA or Chl alone (Fig. 2).

### Effect of APA on autophagy associated markers

**Fig. 3:**
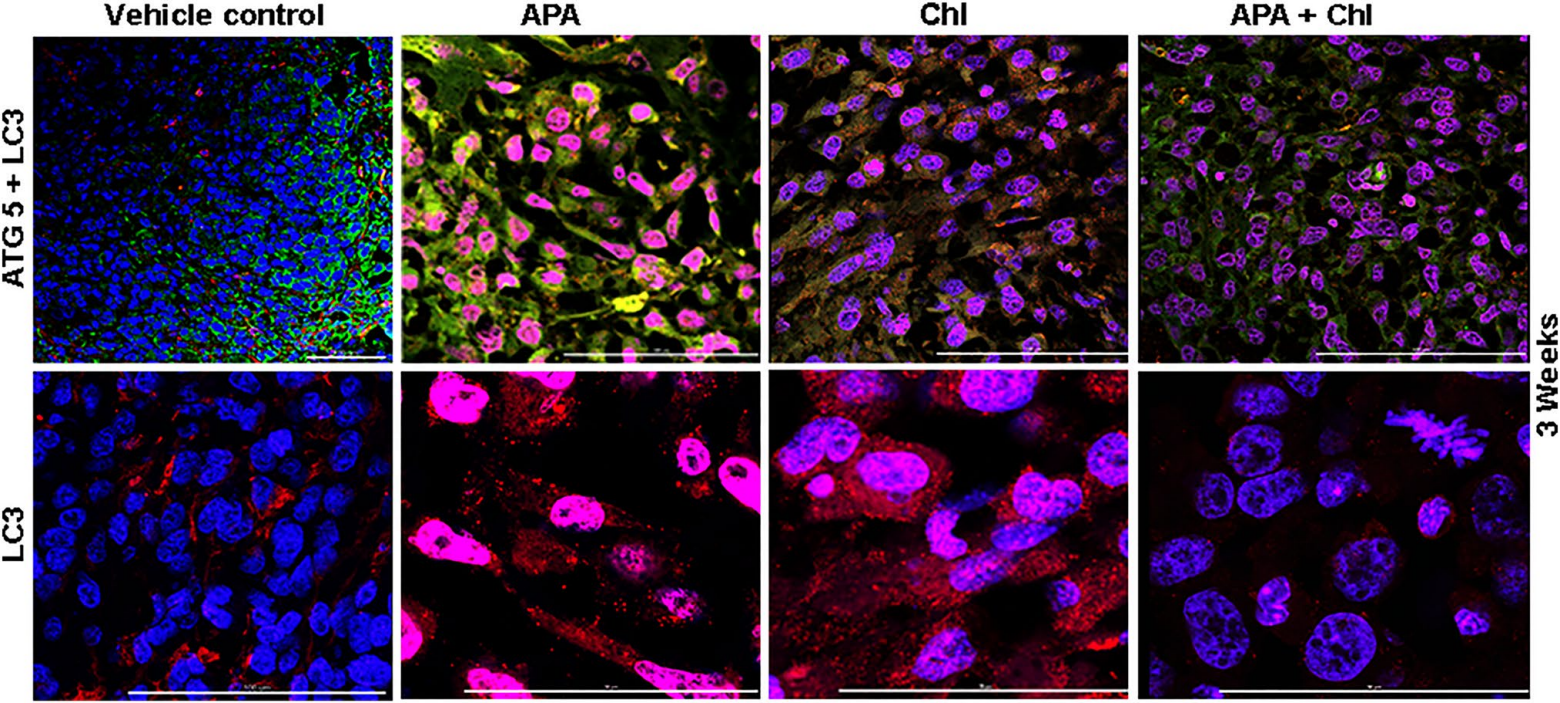
Up-regulation of ATG5 and LC3 in APA-treated animals. Representative immunofluorescent staining of tumor sections from all experimental conditions after 3 weeks of treatments. The green color indicates cytoplasmic expression of ATG5, the LC3 staining in red indicates autophagosome formation. Up-regulation of ATG5 and LC3 punctuation depict high autophagic activity in APA-treated animal tissue sections. Tumor tissue sections combination treated animals showed decreased ATG5 expression. Samples were stained using a Cy3 (red) conjugated secondary antibody or FITC (green) and DAPI (blue, 40,6-diamidino-2-phenylindole)

**Fig. 4:**
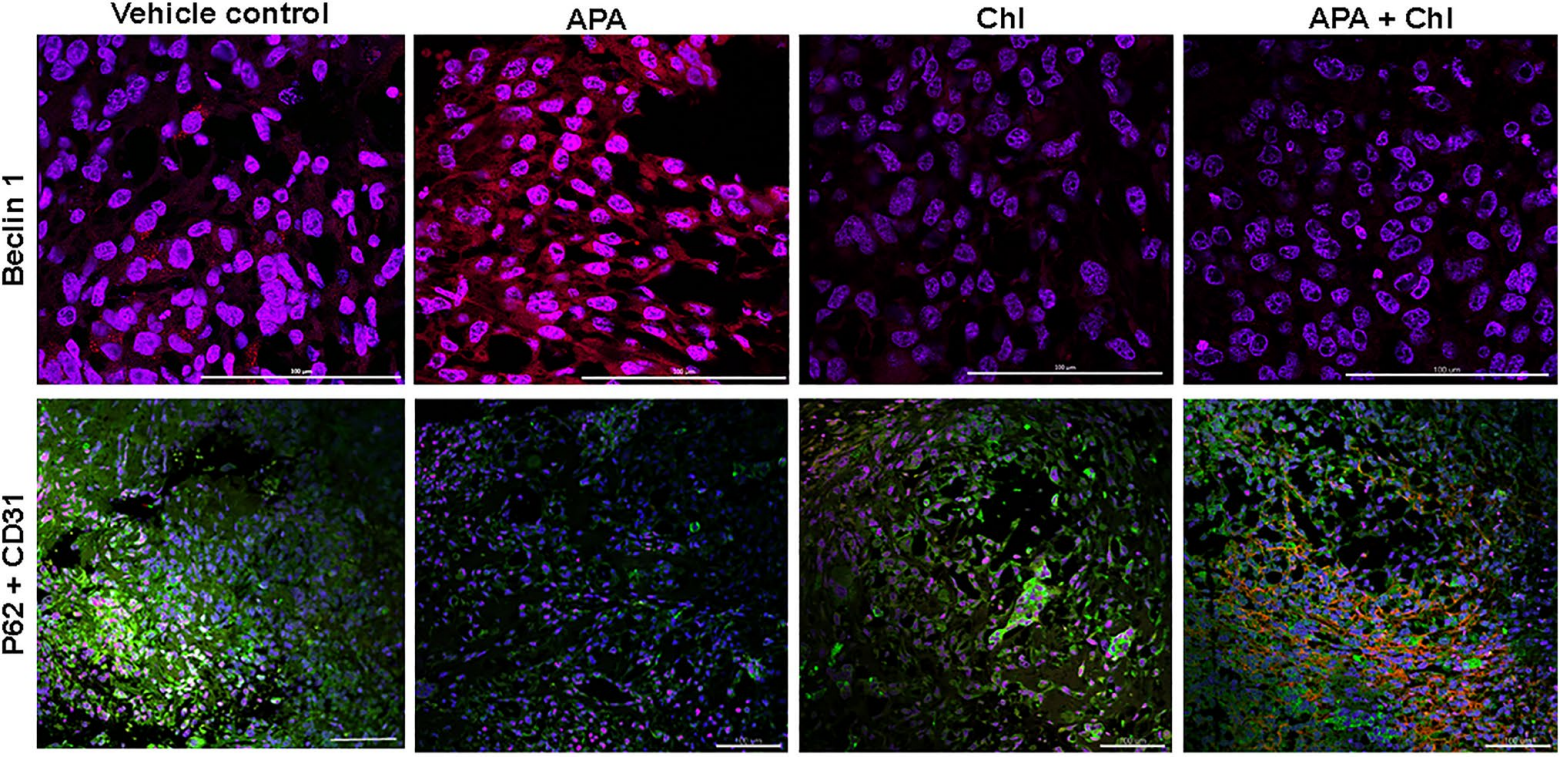
Influence of APA, Chl and combined APA + Chl on Beclin 1 and P62 protein expression. Representative immunofluorescent staining of samples from all experimental conditions after 3 weeks of treatments. Upper panel displays upregulation of Beclin 1(red) in APA-treated animal tissue only. Lower panel shows increased P62 (Green, FITC) and CD31 (red, Cy3) expression in Chl, APA + Chl-treated animals compared to APA-treated animals only. Nuclei were stained with DAPI (blue)

To confirm the qualification of APA and autophagy inhibitors for the use in future therapeutics targeting in clinical trials, the investigation of autophagy specific markers is of importance. Therefore, we evaluated the expression of the main autophagy markers ATG5, Beclin 1 and LC3 in tumor tissue. As assessed by immunofluorescence, vehicle control showed low basal expression levels of ATG5 and a weak diffused LC3 staining (Fig. 3). APA-treated mice showed an increased expression of ATG5 and a punctuated pattern for LC3, confirming the accumulation of autophagosomes upon 3-week treatment. The mice treated with a combination of APA + Chl exhibited a reduced expression of ATG5 and slight LC3 punctuations (Fig. 3, lower panel). Moreover, the Chl-treated mice showed accumulation of LC3 protein, indicating a Chl-induced accumulation of autophagic vacuoles (Fig. 3, lower panel). The observed pattern of expression of Beclin 1 was consistent with the ATG5 results (Fig. 4, upper panel).

Furthermore, immunofluorescent staining with cluster of differentiation 31 (CD31) showed an enhanced vascularization and the presence of endothelial cells in the combination treatment group compared to vehicle control, APA- and Chl-treated groups (Fig. 4). Increased CD31 and reduced autophagy were correlated to an increase in P62 in the combination treatment group.

### Reduction in of the Ki-67 proliferation marker and caspase activation following combination treatment

**Fig. 5:**
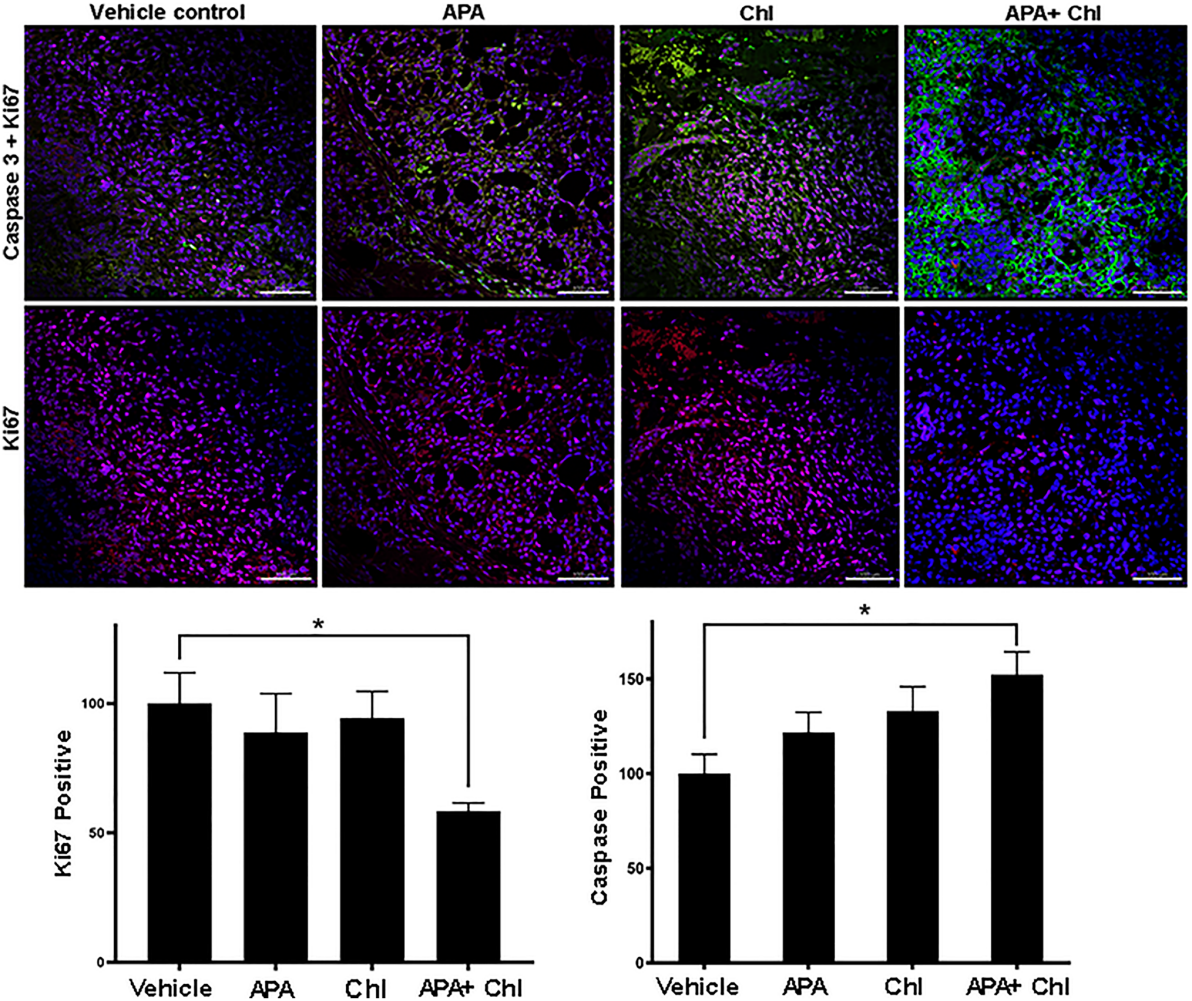
Immunofluorescent colocalization of Ki-67 and caspase 3. Upper panel: Ki-67 (red) and caspase 3 (green) staining of tumor sections after 3 weeks of treatments. Lower panel: Positive Ki-67 immunostaining in the nuclei of tumor cells and cleaved (active) caspase 3 in the cytoplasm of tissue sections were measured and compared to vehicle control (100%). Nuclei were stained with DAPI (blue)

The proliferative capacity of the injected cells was assessed by Ki-67 staining after 3 weeks of treatments. The Ki-67 antibody reacts with a human nuclear antigen that is present only in the nucleus of cells with proliferating capacity. Nuclear staining was detected in all samples. Quantification of Ki-67 fluorescence intensity revealed a significant reduction in APA + Chl (58.3 ± 3.1%)-treated animals compared to vehicle control (100 ± 11.8%), whereas treatment with APA (88.7 ± 14.9%) or Chl (94 ± 2.0%) alone led to a slight reduction of Ki-67 only. These data support the hypothesis that autophagy controls cellular stress and manages also the impact of the APA treatment. Inhibition of autophagy with Chl, therefore, hampers the autophagic defense mechanism and increases the APA efficiency, leading to cell death, apoptosis and induction of cleaved caspase 3 (Fig. 5). Mice treated with APA + Chl showed increased caspase 3 expression (152.3 ± 12.0%) compared to vehicle control (100 ± 10.0%). Increased apoptosis was also observed in animals treated with APA (121.7 ± 10.0%) or Chl (133 ± 12.8%) alone compared to vehicle control. The level of apoptosis appeared to be correlated with the reduction of Ki-67 protein and the increase in cleaved caspase 3 protein expression.

**Fig. 6:**
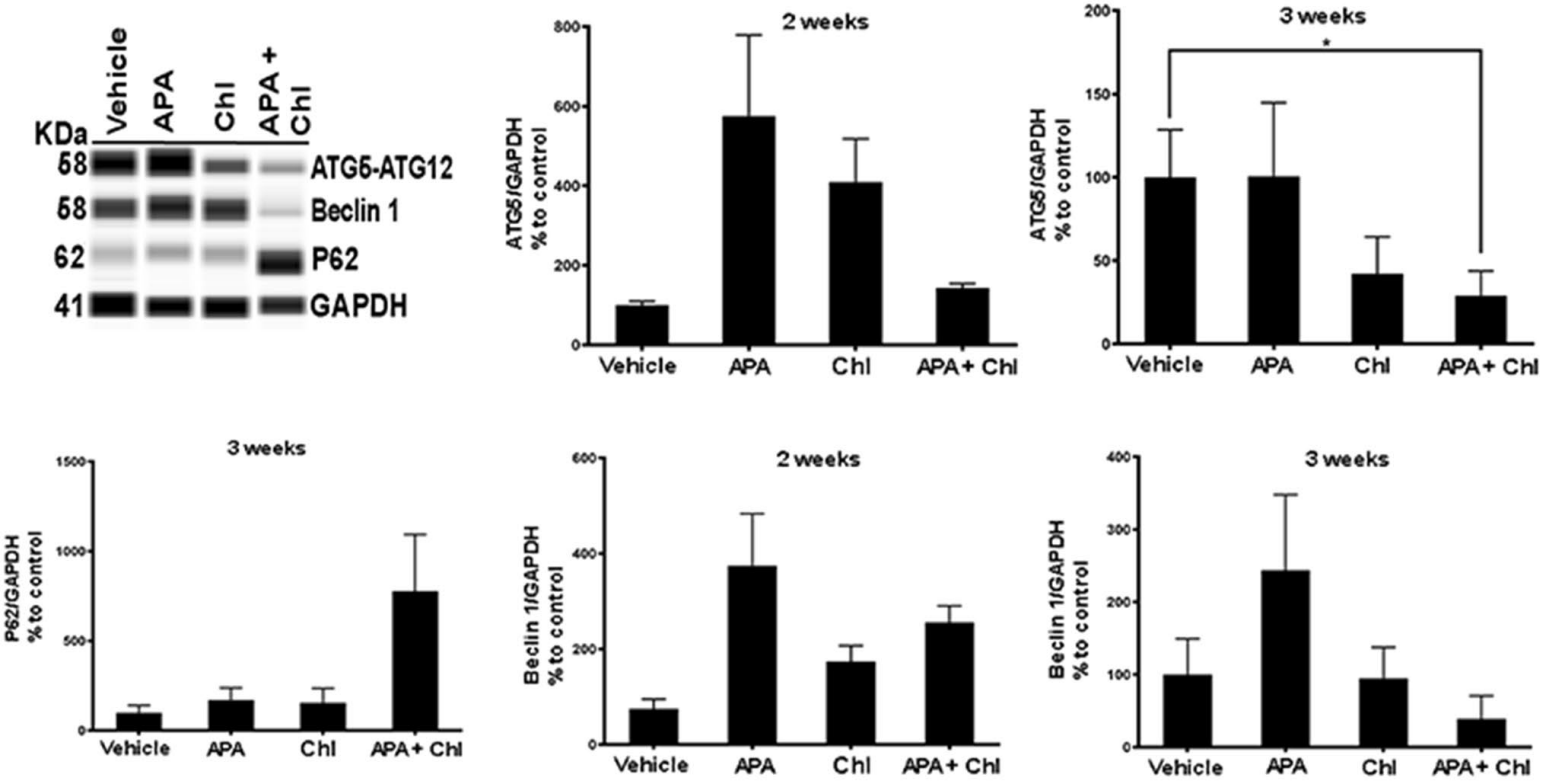
Representative WES immunoblotting image. ATG5 and Beclin 1 protein was increased in animals treated with APA compared to vehicle control and Chl treatments. Mice treated with the combination treatment showed reduction of both ATG5 and Beclin 1 proteins. An increase in P62 protein was observed only in animals with the combination treatment, indicating lowered autophagy in these animals. The protein expression in each sample was normalized to its own GAPDH and analyzed using the Compass software (ProteinSimple). *N* = 5

The involvement of autophagy was confirmed at the protein level by quantitative automated immunoblotting. As shown in Fig. 6, mice receiving APA treatment showed increased levels of ATG5 (112 ± 33%) and Beclin 1 (123.7 ± 10.0%) proteins, i.e., the main regulators of autophagy, when compared to animals treated with vehicle only (100%). Cells treated with Chl showed reduced expressions of ATG5 (51.0% ± 27.4) and Beclin 1 (47% ± 16.0) compared to vehicle control. The combination treatment with APA + Chl led to a significant down regulation of ATG5 (32.53% ± 10.9) and Beclin 1 (35.9% ± 18.0) compared to vehicle control (100%). The reduction of autophagy resulted in the consequential upregulation of ubiquitin-binding protein P62 (345.0% ± 165.4) compared to vehicle control.

## Discussion

The suppression of the AR signaling remains a significant pathway in the therapy of advanced prostate cancer. The present study confirms that cancer cells use autophagy as a survival mechanisms in response to apalutamide (APA) treatment (Eberli et al. 2020). We could demonstrate that autophagy is one of the mechanisms of resistance to APA also in vivo. The combination of APA with the inhibition of autophagy by Chl treatment resulted in enhanced cytotoxicity of PC cells, increased apoptosis and reduction of the xenograft growth in a castrated nude mouse model. Therefore, a combination treatment could hinder the protective effect of autophagy on PC cells and increase APA efficacy, leading to cell death and induction of caspase 3. The upregulation of autophagy as a survival mechanism in CRPC has already been shown in several PC cell lines (Kranzbuhler et al. 2019; Zou et al. 2012; Wu et al. 2010).

In our prior in vitro studies, we have shown, that autophagy is induced as a pro-survival response to multiple cytotoxic therapies, such as APA, abiraterone acetate and EPI-001 in LNCaP, and enzalutamide-resistant LNCaP cells (Kranzbuhler et al. 2019; Eberli et al. 2020; Mortezavi et al. 2019). This induction was associated with an increase in ATG5 and Beclin 1 protein expressions, the main regulators of autophagy in PC cells. In our present study, we have followed the same strategy and used APA (10 mg/kg/day) and Chl (10 mg/kg/day) alone and in combination in a mouse xenograft model of human CRPC. Our results are consistent with a previously published animal study, demonstrating the safety and efficacy of APA (10 mg/kg/day) combined with a dose-dependent tumor regression that is superior to bicalutamide or enzalutamide (4). Consistent with our in vitro data, the main proteins involved in autophagosome formation, namely ATG12-ATG5 (ATG5) and Beclin 1, were upregulated upon single treatment with APA (Eberli et al. 2020). We also demonstrated that a treatment with APA alone induced LC3 (microtubule-associated protein 1 light chain 3-phosphatidylethanolamine (PE) system) and decreased the expression of P62 in the tumor xenograft tissue. This is in line with our prior reports where we demonstrated an increased LC3 localization and a decrease in P62 with induction of autophagy in APA-treated cells (Eberli et al. 2020; Saleem et al. 2012).

Increasing evidence from preclinical models suggests that inhibition of autophagy increases cytotoxicity in combination with several anticancer drugs (Kaini and Hu 2012; Boutin et al. 2013). Autophagy inhibitors, such as chloroquine and 3-methyladenine, are used to sensitize several different cancer cells to different anti-cancer drugs, such as cisplatin or tamoxifen, as well as to radiation therapies (Wu et al. 2010; Amaravadi et al. 2007; Apel et al. 2008).

Therefore, we investigated the impact of APA in combination with an agent such as chloroquine (Chl). This drug, which is approved by the FDA (food and drug administration), is a known inhibitor of autophagy, initially used to prevent and treat malaria (Rubinsztein et al. 2007). It blocks autophagy at later stages in the autophagic progression by interfering with lysosome acidification and by impairing autophagosome degradation (Rubinsztein et al. 2007). Chl alone or in combination with different anti-cancer drugs or chemotherapeutics has been used in numerous clinical trials for the treatment of various cancers and was reported to be well-tolerated (Wolpin et al. 2014; Rangwala et al. 2014; Rosenfeld et al. 2014; Boone et al. 2015). The safety of Chl in combination with chemotherapeutic taxanes was evaluated in a phase II clinical study of breast cancer. This combination therapy was well tolerated and effective in patients with locally advanced or metastatic breast cancers. The clinical use of Chl in combination with the Akt inhibitor MK2206 for the treatment of patients with advanced solid tumors such as PC is currently under investigation (Phase I, Study identifier: NCT01480154). In our study, we further show evidence for increased cytotoxicity using a combination treatment of APA + Chl in our mouse model. Mammalian cells and tissues lacking autophagy have increased levels of ubiquitin and P62 (Bjorkoy et al. 2005; Tanida and Waguri 2010). Therefore, an increase in P62 protein expression confirms that autophagy is blocked in the combination treated mice. Furthermore, this led to an increase in caspase 3 protein expression and reduction in Ki-67 protein. As a marker of cell proliferation, Ki-67 is measured in prostate tumor tissues as an additional prognostic marker (Ojea Calvo et al. 2004; Hammarsten et al. 2019). Therefore, the reduction of Ki-67 protein in the combination treated mice is an indicator of hampered cell proliferation, which correlated with an increase in cleaved caspase 3 protein.

The efficacy of APA, a competitive inhibitor of AR, was evaluated in a study including men with non-metastatic CRPC who were at high risk for the development of metastasis. In the group receiving APA treatment, the risk of metastasis or death was 70% lower and the median metastasis-free survival was extended by more than 2 years compared to the placebo group (7). A further phase III study (SPARTAN) assessed the benefit of APA for the overall survival of none metastatic CRPC patients. In the APA-treated group, the risk of death decreased by 22% and the median overall survival increased by 14 month compared to placebo (Smith et al. 2020).

Despite advances in the development of drugs for the treatment of advanced prostate cancer, the exact mechanism of resistance by which the targeted cells might escape is still unknown and requires further research.

## Conclusion

In this study, we provide preclinical data demonstrating that targeting autophagy in combination with an APA treatment may enhance tumor suppressive effects in CRPC. Autophagy reduces treatment stress and promotes cell survival of tumor cells, which ultimately may allow them to develop other ways to resist anti-androgen therapies. A novel safe and efficient way to overcome resistance mechanisms might be the combination therapy of APA with autophagy modulators such as chloroquine. A combination therapy that simultaneously targets the androgen receptor axis and autophagy may maximize the therapeutic effect in CRPC patients in future clinical applications.

## Abbreviations

APA: Apalutamide
AR: Androgen receptor
ATG5: Autophagy-related 5 protein
Chl: Chloroquine
CRPC: Castration-resistant prostate cancer
CY3: Cyanine-3
DAPI: 4′,6-Diamidino-2-phenylindole
FITC: Fluorescein isothiocyanate
PC: Prostate cancer
i.p.: Intraperitoneal
